# Presurgical Nasoalveolar Molding for Correction of Cleft Lip Nasal Deformity: Experience From Northern India

**Published:** 2010-07-23

**Authors:** Brijesh Mishra, Arun K. Singh, Javed Zaidi, G. K. Singh, Rajiv Agrawal, Vijay Kumar

**Affiliations:** ^a^Department of Plastic Surgery; ^b^Department of Orthodontics, Chhatrapati Shahuji Maharaj Medical University (Erst while King Georges Medical College), Lucknow, Uttar Pradesh, India

## Abstract

**Context:** The cleft lip type nasal deformity presents one of the most complex surgical challenges. The long-term postoperative results are still not satisfactory despite an emphasis on primary nasal correction. This is attributed to tissue memory and healing. Nasoalveolar molding is used effectively to reshape the nasal cartilage and to mold the maxillary arch before cleft lip repair. **Aims:** This study was undertaken to evaluate the role of presurgical nasoalveolar molding in correction of cleft lip nasal deformity for patients with unilateral and bilateral clefts of the lip. **Settings and Design:** Twenty-three cases of clefts of lip and palate with nasal deformity were subjected to present study from May 2004 to May 2006. These cases were initially treated on outpatient basis, and they were admitted at the time of operation. All of these patients were children of less than 1 year of age, belonging to north Indian population. **Material and Methods:** Study consisted of patients of cleft lip and palate who were given presurgical nasoalveolar splints at early age. Lip repair was done after at least 2 months of molding. These patients along with control group (without presurgical nasoalveolar molding) were followed up for 1 year. Measurements were taken at different intervals in study over dental cast and on patients. Data obtained from comparison of 2 groups were analyzed using “MSTAT” analysis software (developed by Dr Russel Freed, Professor & Director, Crop & Soil Sciences Department, Michigan State University, East Lansing, Michigan). **Results:** In our study, we found that nostril height was more in patients of experimental group (*P* = .18), while nostril width and alar perimeter were not changed significantly. Children with nasoalveolar molding had significant lengthening of columella (*P* = .02). Patients of unilateral cleft lip had more reduction in alveolar gap (*P* = .08) than bilateral group (*P* = .15). **Conclusions:** Nasoalveolar molding can be a useful adjunct for treatment of cleft lip nasal deformity. It is a cost-effective technique that can reduce the number of future surgeries such as alveolar bone grafting and secondary rhinoplasties.

The management of cleft patients has evolved dramatically in recent years. Outcome is improving because of better surgical techniques, timing, and incorporation of procedures like presurgical orthopedics.

Presurgical infant orthopedics was first introduced by McNeil^1^ in 1950. Since then, techniques are changing and so are the results. Active molding and repositioning of the nasal cartilages take advantage of the plasticity of cartilage in the newborn infant.^2^,^3^

In the last decade, it has been shown that correction of nasal deformity by stretching of the nasal mucosal lining, and achievement of nonsurgical columella elongation can be combined with molding of the alveolar process in cleft patients.^4^,^5^

Multiple reports have come from around the world about efficacy and utility of nasoalveolar molding with different opinions; however, studies on Indian populations are lacking. Objective of this study was to evaluate the role of nasoalveolar molding for correction of cleft lip nasal deformity in Indian patients and to see efficacy of molding in different age groups.

## SUBJECTS AND METHODS

Twenty-three cases of clefts of lip and palate with nasal deformity were subjected to present study from May 2004 to May 2006. These cases were initially treated on outpatient basis, and they were admitted at the time of operation. All of these patients were children of less than 1 year of age, belonging to north Indian population.

Their parents were explained about the cleft deformity and various stages of treatment. They were specially told about the procedure of nasoalveolar molding, the technique, requirement for periodic checkups, and sequential correction. Local patients or patients from nearby places who consented for weekly follow-ups were chosen for study.

### Preparation of splint

The impression was obtained with the infant fully awake, in prone position without anesthesia. Before impressions, child was kept nil orally for about 2 hours. Impressions were taken on dental chair with child in the lap of his or her parents. Impression should be taken very carefully and is always done after insuring the availability of anesthesia team.

First, the impression tray was checked in the mouth of patient. After selection of a proper size tray, alginate paste was made, loaded in the tray, and inserted in the mouth. Soon after this, alginate paste was applied over the plate by hand up to root of the nose. Child's lower jaw was pulled down, and precautions were taken to avoid falling of impression material into oral cavity. After some time (15-20 s), this nose, lip, and alveolus negative impression was removed in a single piece. Oral and nasal cavities were inspected for any remaining particles.

After impression, a dental stone cast was made by filling it with paste of dental stone material. It was allowed to fix. Dental stone model was made for purpose of measurements and fabrication of appliance. These dental stone casts were labeled with patient's name, age/sex, and date.

**Figure 1:**
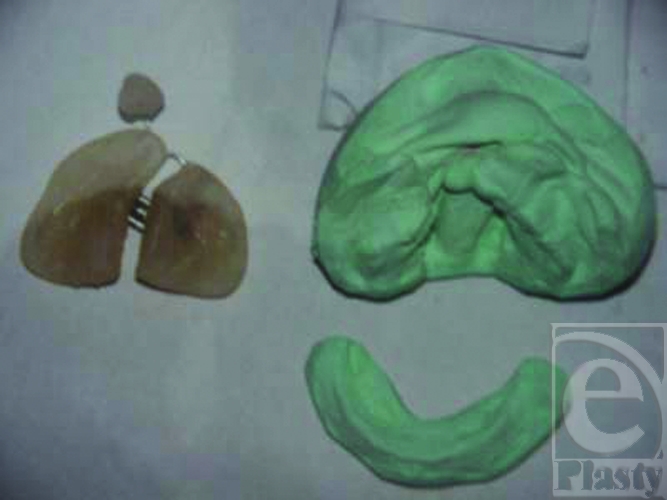
Dental model and fabrication of nasoalveolar moulding splint.

A conventional molding plate was fabricated on the maxillary cast using clear acrylic resin with a nasal stent wire passed from it going superiorly toward nose. The tip of wire was covered with hard and then soft acrylic. At the active tip of nasal stent, the acrylic was covered with a thin layer of soft denture lining material to insure that tissue irritation does not occur when pressure is applied for nasoalveolar molding (Fig 1).

After the nasoalveolar molding plate was ready, it was examined for any rough areas. Plate was handed over to parents, and they were explained about maintaining oral hygiene, cleaning, insertion, and removal of plate.

Patients were called at weekly intervals to gradually change the direction of nasal wire.

At every visit, local area was examined for any ulceration or pressure points. Measurements of different nasal parameters and alveolus were taken on prepared dental stone model as well as on patients directly with the help of thread and artery forceps, and were recorded. It was done for the purpose of accuracy. Measurements on patients were matched with measurements on dental models, and they were found to be almost similar.

**Figure 2:**
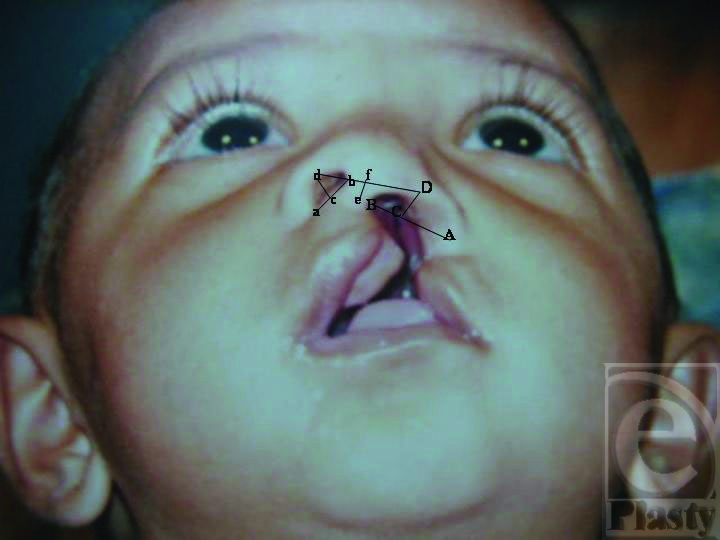
Measurements before lip repair.

**Figure 3:**
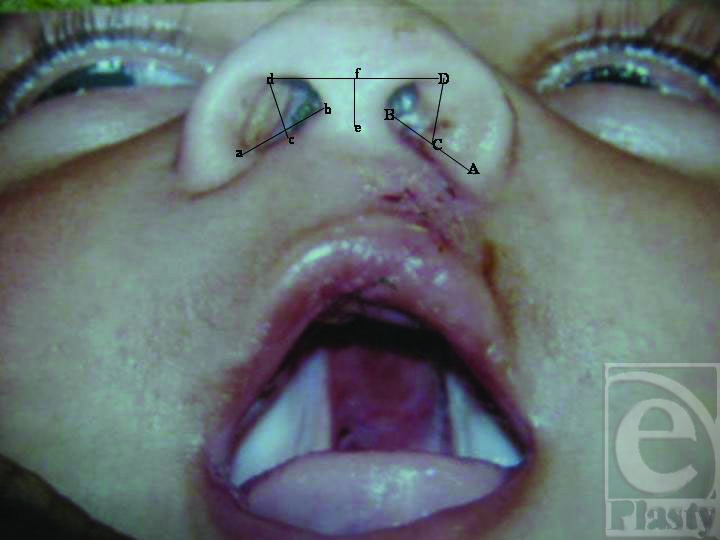
Measurements after lip repair.

Following Reference points were used for different measurements (Figs 2 and 3):

- alar base noncleft side
- columellar base noncleft side
- midpoint of a-b, centre of floor of the nose
- the highest point on the alar rim noncleft side
- midpoint at the base of columella
- the highest point in the midline of columella

- alar base cleft side
- columellar base cleft side
- midpoint of A-B, centre of floor of the nose
- the highest point on the alar rim noncleft side

These measurements included the following:

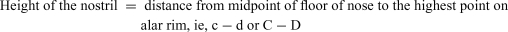

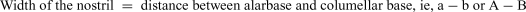

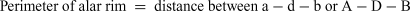

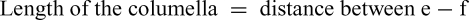

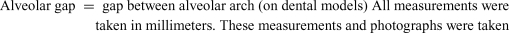











– At the time of first presentation before beginning of nasoalveolar molding (NAM).

– At the time of operation of lip repair.

– At 1-year follow-up or when these patients came for palatal repair.

All these patients were called for operation after 2 to 3 months of molding. Patients were operated in similar settings with a fixed team of plastic surgeon and assistants. All patients were operated by Randall Tennison's method of lip repair with no primary nasal correction done at the time of lip repair. In these patients only septum was freed from ant nasal spine. In no case, nasal incisions, domal separation, undermining of skin, or bolsters stitches were given. After operation, stitches were removed on fifth day and, patients were discharged. They were called in cleft clinics for monthly follow-ups.

Patients with or without nasoalveolar molding were called at 1 year of age for palatal repair. Control group was randomly chosen from patients without nasoalveolar molding who consented for dental models and measurements. All of these patients had no presurgical nasoalveolar molding, and these were operated by same team of surgeons using the similar technique of repair with no primary nasal correction. Dental models were made for these patients, and measurements were taken on dental models as well as patients.

### Statistical evaluation

The data obtained were subjected to statistical analysis. Statistical analysis was done with “MSTAT” analysis software (Michigan State University, East Lansing, Michigan). Mean (SD) was calculated for all groups. Paired *t* test and unpaired *t* test were used to test the significance of change in variables and difference in 2 groups. Values are presented are mean (SD) and percentage.

## RESULTS

**Figure 4:**
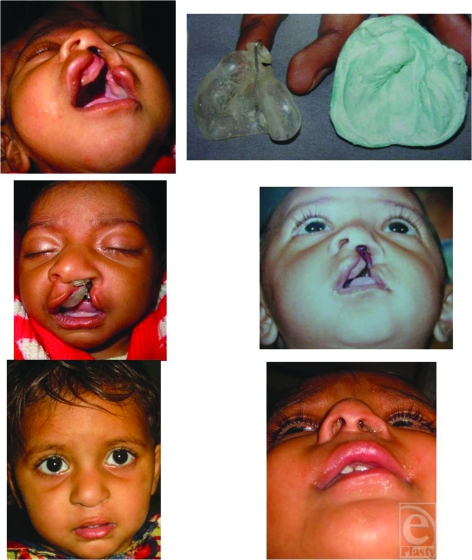
Case 1 : Pt of left unilateral cleft lip and palate before moulding, after moulding and after lip repair.

**Figure 5:**
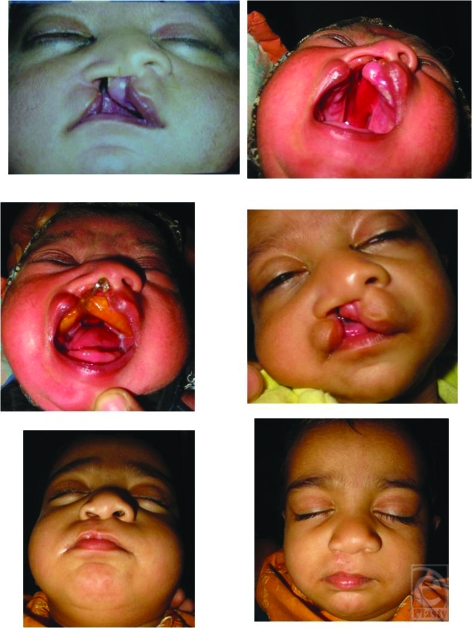
Case 2 : Pt of right unilateral cleft lip and palate before moulding, after moulding and after lip repair.

**Figure 6:**
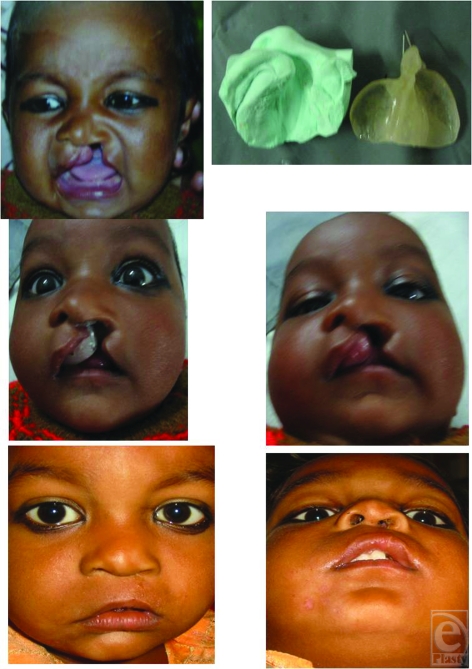
Case 3 : Pt of left unilateral cleft lip and palate before moulding, after moulding and after lip repair.

**Figure 7:**
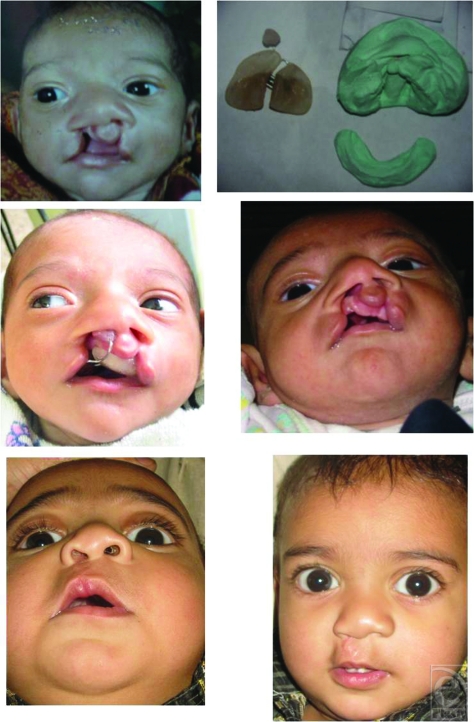
Case 4 : Pt of bilateral cleft lip and palate before moulding, after moulding and after lip repair.

**Figure 8:**
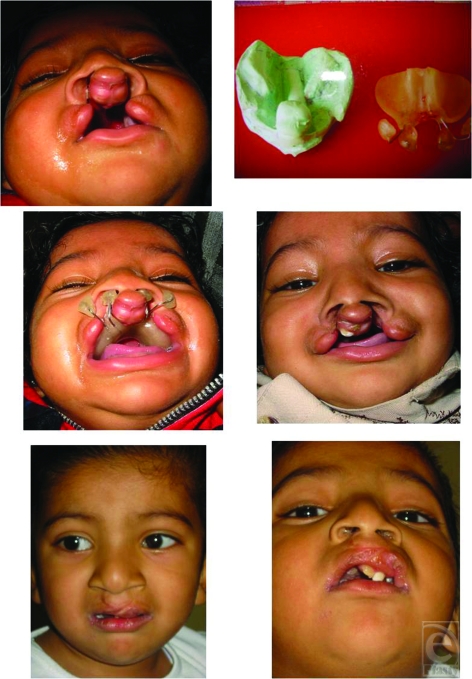
Case 5 : Pt of bilateral cleft lip and palate before moulding, after moulding and after lip repair.

These 23 patients of cleft lip nose deformity of experimental group were of age ranging from 10 to 360 days. The experimental group had 17 patients of unilateral cleft nose deformity and 6 patients of bilateral cleft nose deformity. The duration of the NAM ranged from 2 to 3 months (average 2 months 10 days) (Figs 4–8).

In unilateral clefts difference in nostril height on cleft side was higher in experimental group than control group (*P* = .18), while noncleft side in both groups were almost similar (*P* = .85). In bilateral clefts nostril height was increased in both sides in experimental group than control group (*P* = .30 for both sides) (Table 1).

Columellar length was found significantly higher for cases both in unilateral and bilateral clefts (*P* = .05 for both unilateral and bilateral cases). Relative comparison of columellar lengthening shows that lengthening of columella is significantly higher in unilateral cases than bilateral cases (*P* = .02) (Table 2).

After 1 year of lip repair, alveolar gap was found higher in control group both in unilateral (*P* = .08) and bilateral cases (right, *P* = .15; left, *P* = .15). Relative comparison within the experimental group shows that presence of alveolar gap is higher in bilateral cases on both sides in comparison with gap in unilateral cases (right, *P* = .45; left, *P* = .15) (Table 3).

Patients were divided in 4 groups according to their age of starting the NAM. Maximum change was observed in first 2 groups. Infant up to 6 weeks had maximum effect of the NAM, especially, on cleft side, that is, 72% increase from pretreatment level. This increase is 66.7% in group II, 38% in group III, and 47% in group IV. It signifies that molding is most effective if done in early age groups (Table 4).

Gain in columellar length was maximum in group I (42.1%), and gain in length decreases as the age of starting of molding increases (30% in group II, 26% in group III, and 19.1% in group IV). Reduction in alveolar gap is maximum in group I (50.1). Change in alveolar gap in other group was 36% in group II, 33.3% in group III, and 50% in group IV (Table 5).

## DISCUSSION

Bardach and Cutting^6^ in 1990 described the NAM by acrylic intraoral molding plate with a nasal stent of acrylic, rising from the labial vestibular flange. Similar procedure was described by Hotz and Gnoinski^7^ as Zurich type molding plate, but only for alveolus. Cutting and Bardach gradually added small amount of acrylic resin to lift the alar dome cartilage on the cleft side to achieve normal elevation and symmetry.

Our appliance is nearly same except that we used an orthodontic wire covered by an acrylic bulb to give pressure for active molding. Our appliance is more cost-effective, because it does not need any further addition of acrylic every week, only wire angle is increased a bit to increase the pressure exerted.

Matsuo and Hirose^2^,^3^ showed role of preoperative molding in changing the cartilage memory of deformed nasal cartilage, because these cartilages have higher amount of hyaluronic acid, which gradually diminishes after few months of birth.

In our study we found that changes because of molding were most significant in the age group of birth to 6 weeks, and they were better in first 3 months of life. It shows that beneficial effect of molding is maximum in the youngest children.

Maull and colleague,^5^ Cutting and colleague,^8^ and Grayson and colleague,^9^ studied long-term effects of the NAM on 3-dimensional nasal shape in unilateral clefts by using nasal cast of the subjects. They scanned these casts in 3 dimensions, and a numerical asymmetry score was determined. The mean asymmetry index for the NAM group was 0.74, and for the control group it was 1.21. This difference was statistically significant (*P* < .05). They concluded presurgical NAM significantly increases the symmetry of the nose.

Grayson and Maull^10^ and Cutting and colleague^11^ evaluated the financial impact of 2 treatment approaches to the unilateral cleft alveolus. They compared NAM, and gingivoperiosteoplasty at the time of lip repair with the traditional approach of lip repair followed by secondary alveolar bone graft. Of the 16 patients treated by NAM, gingivoperiosteoplasty, lip repair, and primary nasal repair, 10 required no further treatment of the unilateral cleft alveolus; 6 patients required secondary alveolar bone graft.

Our study also shows a trend of reduced alveolar gap in experimental patients after NAM that may lead to avoidance of surgery in future like alveolar bone grafting and secondary rhinoplasties.

Da Silveira et al^12^ have also described a similar appliance with metallic wire and found it to be useful and more easy to manipulate.

Liou EJ et al^13^ assessed 25 infants for the progressive changes of nasal symmetry, growth, and relapse by direct linear measurements on photographs and concluded that the nasal asymmetry was significantly improved after nasoalveolar molding and was further corrected to symmetry after primary cheiloplasty. After the primary cheiloplasty, the nasal asymmetry significantly relapsed in the first year postoperatively and then remained stable and well afterward. The relapse was the result of a significant differential growth between the cleft and noncleft sides in the first year postoperatively.

Pai BC et al^14^ in their study concluded that molding improved symmetry of the nose in width, height, and columella angle, as compared with their presurgical status. There was some relapse of nostril shape in width (10%), height (20%), and angle of columella (4.7%) at 1 year of age.

In our study the change in nostril height on cleft side of nostril was significant in experimental group at the time of lip repair (*P* = .001), and at 1 year of age it was slightly less significant (*P* = .18) when compared with control group, while there was insignificant change observed in nostril width and nasal alar perimeter. Nostril width was slightly increased in bilateral cases.

Doruk and Kilic^15^ suggested extra oral nasal molding appliance for presurgical NAM in newborns, but in our experience an extra oral appliance is very difficult to retain on these infants and compliance was poor in such cases.

Deng et al^16^ observed clinical effect of presurgical NAM in infants with complete cleft lip and palate. After 108 to 152 days of therapy, the average width of alveolar cleft decreased by 5.3 mm in 26 patients with unilateral cleft lip and palate. Nasal profile was improved in 76% of cases. In 12 patients with bilateral cleft lip and palate, the average width of left cleft decreased by 4.7 mm and that of the right decreased by 4.2 mm. The distance between right and left cleft increased by 5.1 mm. Nasal profile was improved in 66% of cases.

Our findings are correlating closely with their studies showing better nostril height and better profile in unilateral cases then bilateral cases.

**Figure 9:**
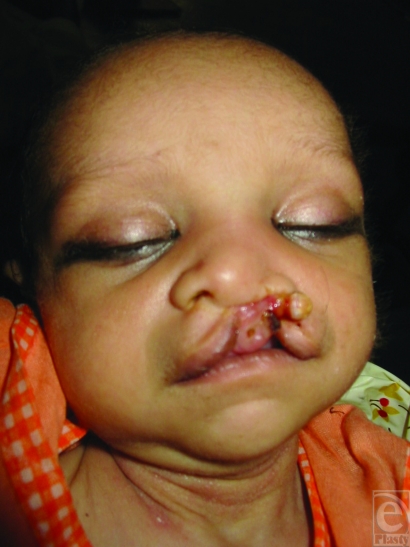
Ulceration after application of molding appliance.

Ziai, Mandana et al^17^ described natal/neonatal teeth in an article. They showed that the teeth interfered with the fabrication and application of the NAM appliance, they were removed, and the NAM device was placed without difficulty. We also encountered a patient who returned after 1 week with a small swelling over the margin of alveolar cleft. Molding plate was withdrawn, inflamed swelling subsided in a week but slight elevation remained. Parents refused for further molding treatment and patient was operated (Fig 9).

We accept the limitations of present study in terms of small sample size, variation in sample size, and smaller follow-up period. This study involves periodic clinic visits, patients need to wait for impression, fabrication of cast, plates and measurements. This is often not possible for patients of rural and remote areas. Study was started with much wider patient base, but many of them did not come back. Mostly, local patients were included for study for obvious reasons. Such studies require educated parents, dedicated paramedics staff, and infrastructure for better reception and effective time management for these patients. All of the factors are the potential causes for limitation of our study. It will definitely be better to have a larger data from many centers with longer follow-ups to produce more scientifically justified reports.

## CONCLUSION

Nasoalveolar molding can be useful adjunct for treatment of cleft lip nasal deformity. It is possible to incorporate presurgical NAM at centers where basic plastic surgery services and support of orthodontist/prosthodontist is present. It can prove to be a cost-effective technique by reducing number of future surgeries in cleft patients. Studies with wider patient base and longer follow-ups are needed for definitive results.

## Figures and Tables

**Table 1:** Comparison of nostril height at 1-year follow-up after lip repair

		Cases with NAM, mean (SD), mm	Controls without NAM, mean (SD), mm	*t*	*P*
Unilateral cases (N = 17)	Noncleft	6.000 (0.9354)	6.0588 (0.9824)	0.18	.85
	Cleft	5.4412 (0.8993)	5.0000 (0.9683)	1.38	.18
Bilateral cases (N = 6)	Right	5.0833 (1.1583)	4.2500 (1.4405)	1.10	.30
	Left	4.8333 (1.2910)	4.0000 (1.4491)	1.05	.30

Abbreviation: NAM, nasoalveolar molding.

**Table 2:** Comparison of length of columella at 1-year follow-up after lip repair

	Cases with NAM, mean (SD), mm	Controls without NAM, mean (SD), mm	*t*	*P*
Unilateral cases (N = 17)	5.2647 (0.7524)	4.5882 (0.8703)	2.42	<.05
Bilateral cases (N = 6)	4.3333 (0.5164)	3.4167 (0.6646)	2.67	<.05

Abbreviation: NAM, nasoalveolar molding.

**Table 3:** Comparison of presence of alveolar gap at 1-year follow-up after lip repair

		Alveolar gap cases with NAM, mean (SD), mm	Alveolar gap controls without NAM, mean (SD), mm	*t*	*P*
Unilateral cases (N = 17)		1.5294 (1.6999)	2.7059 (1.7946)	1.96	.08
Bilateral cases (N = 6)	Right	2.1667 (1.8348)	3.6667 (1.2111)	1.67	.15
	Left	2.1667 (1.5055)	4.080 (1.4142)	1.58	.15

Abbreviation: NAM, nasoalveolar molding.

**Table 4:** Analysis of nostril height in unilateral cases of experimental group according to age of starting of nasoalveolar molding

Subgroup age	Pretreatment, mean (SD), mm	Preoperative, mean (SD), mm	Change, mean (SD), mm	%change
Group I (birth-6 wk)				
Noncleft	3.333 (0.7528)	4.4167 (0.6646)	1.0833 (0.5845)	32.5
Cleft	2.6661 (0.7528)	4.5853 (0.6646)	1.9167 (0.7360)	72.0
Group II (7 wk-3 mo)				
Noncleft	3.667 (0.6055)	4.500 (0.9487)	0.8333 (0.9309)	22.73
Cleft	2.7500 (0.2739)	4.5833 (1.0206)	1.8333 (1.0328)	66.7
Group III (4 mo-6 mo)				
Noncleft	4.1667 (0.7600)	4.8333 (0.7638)	0.6667 (0.2887)	16.0
Cleft	3.500 (0.5000)	4.8333 (0.7638)	1.3333 (0.2887)	38.0
Group IV (7 mo-1 y)				
Noncleft	6.000 (2.8284)	6.2500 (2.4749)	0.2500 (0.3536)	4.2
Cleft	4.2500 (2.4749)	6.2500 (2.4749)	2.0000 (0.4210)	47.1

**Table 5:** Analysis of columellar length and alveolar gap in unilateral cases of experimental group according to age of starting of nasoalveolar molding

Subgroup age	Pretreatment, mean (SD), mm	Preoperative, mean (SD), mm	Change, mean (SD), mm	%change
Group I (birth-6 wk)				
Col length	3.1667 (0.7528)	4.500 (0.8367)	1.3333 (0.5164)	42.1
Alv gap	6.8333 (2.1370)	3.4167 (1.4287)	3.4167 (1.0206)	50.1
Group II (7 wk-3 mo)				
Col length	3.3333 (0.6055)	4.333 (0.8165)	1.000 (0.8367)	30
Alv gap	7.500 (3.9370)	4.8333 (1.3292)	2.6667 (3.4448)	36
Group III (4 mo-6 mo)				
Col length	3.8333 (0.2887)	4.8333 (0.7683)	1.00 (0.50)	26.1
Alv gap	4.0000 (6.9282)	2.6667 (4.6182)	1.333 (2.3094)	33.3
Group IV (7 mo-1 y)				
Col length	5.2500 (2.4749)	6.25 (2.4749)	1.000 (0.000)	19.1
Alv gap	4.000 (5.6549)	2.00 (2.8284)	2.000 92.8284)	50.0

Abbreviations: Alv gap, alveolar gap; Col length, columellar length.
